# Structural Features of Amyloid Fibrils Formed from the Full-Length and Truncated Forms of Beta-2-Microglobulin Probed by Fluorescent Dye Thioflavin T

**DOI:** 10.3390/ijms19092762

**Published:** 2018-09-14

**Authors:** Anna I. Sulatskaya, Natalia P. Rodina, Dmitry S. Polyakov, Maksim I. Sulatsky, Tatyana O. Artamonova, Mikhail A. Khodorkovskii, Mikhail M. Shavlovsky, Irina M. Kuznetsova, Konstantin K. Turoverov

**Affiliations:** 1 Laboratory of Structural Dynamics, Stability and Folding of Proteins, Institute of Cytology of the Russian Academy of Science, Tikhoretsky ave. 4, St. Petersburg 194064, Russia; ansul@mail.ru (A.I.S.); natalia240994@gmail.com (N.P.R.); m_sulatsky@mail.ru (M.I.S.); imk@incras.ru (I.M.K.); 2 Department of Molecular Genetics, Institute of Experimental Medicine, Pavlov str. 12, St. Petersburg 197376, Russia; ravendoctor@mail.ru (D.S.P.); mmsch@rambler.ru (M.M.S.); 3 Chair of Medical Genetics, North-Western State Medical University named after I.I. Mechnikov, Piskarevskij prospect 47, St. Petersburg 195067, Russia; 4 Research Center of Nanobiotechnologies, Peter the Great St. Petersburg Polytechnic University, Polytechnicheskaya 29, St. Petersburg 195251, Russia; artamonova@nanobio.spbstu.ru (T.O.A.); khodorkovskii@mail.ru (M.A.K.); 5 Institute of Physics, Nanotechnology and Telecommunications, Peter the Great St. Petersburg Polytechnic University, Polytechnicheskaya 29, St. Petersburg 195251, Russia

**Keywords:** beta-2-microglobulin (β2M), β2M truncated forms, dialysis-related amyloidosis (DRA), amyloid fibrils, thioflavin T (ThT), equilibrium microdialysis, binding parameters

## Abstract

The persistence of high concentrations of beta-2-microglobulin (β2M) in the blood of patients with acute renal failure leads to the development of the dialysis-related amyloidosis. This disease manifests in the deposition of amyloid fibrils formed from the various forms of β2M in the tissues and biological fluids of patients. In this paper, the amyloid fibrils formed from the full-length β2M (β2m) and its variants that lack the 6 and 10 N-terminal amino acids of the protein polypeptide chain (ΔN6β2m and ΔN10β2m, respectively) were probed by using the fluorescent dye thioflavin T (ThT). For this aim, the tested solutions were prepared via the equilibrium microdialysis approach. Spectroscopic analysis of the obtained samples allowed us to detect one binding mode (type) of ThT interaction with all the studied variants of β2M amyloid fibrils with affinity ~10^4^ M^−1^. This interaction can be explained by the dye molecules incorporation into the grooves that were formed by the amino acids side chains of amyloid protofibrils along the long axis of the fibrils. The decrease in the affinity and stoichiometry of the dye interaction with β2M fibrils, as well as in the fluorescence quantum yield and lifetime of the bound dye upon the shortening of the protein amino acid sequence were shown. The observed differences in the ThT-β2M fibrils binding parameters and characteristics of the bound dye allowed to prove not only the difference of the ΔN10β2m fibrils from other β2M fibrils (that can be detected visually, for example, by transmission electron microscopy (TEM), but also the differences between β2m and ΔN6β2m fibrils (that can not be unequivocally confirmed by other approaches). These results prove an essential role of N-terminal amino acids of the protein in the formation of the β2M amyloid fibrils. Information about amyloidogenic protein sequences can be claimed in the development of ways to inhibit β2M fibrillogenesis for the treatment of dialysis-related amyloidosis.

## 1. Introduction

Beta-2-microglobulin (β2M) is a protein with a molecular weight of 11.8 kDa that is composed of 99 amino acid residues. It is produced in all the nucleated cells of the human organism and it plays an important role in the cellular immunity. β2M facilitates the successful folding and the cell surface exposure of the major histocompatibility complex class I molecules [1,2]. Normally, β2M concentration in blood plasma is approximately 1–3 μg/mL, while about 2–4 mg/kg of the protein is synthesized daily in the body, and its half-life is about 2.5 h [3,4,5]. Approximately 95% of the β2M elimination occurs through glomerular filtration (with subsequent reabsorption and intracellular proteolysis in the proximal tubules), thus its concentration in blood plasma is directly related to the kidneys functioning. The β2M levels in chronic renal failure may increase by 60 times due to a significant (10–15 times) increase in the protein excretion time [5,6]. Large amounts of β2M can been found in the urine of patients with impaired nephrotic reabsorption of proteins from the primary filtrate [7]. During the prolonged hemodialysis therapy that is needed to clean the blood of patients with severe kidney disease, the β2M concentration in blood plasma is constantly much higher than normal. The continuous persistence of high β2M concentrations is considered to be the main reason for the appearance of abnormal protein conformations and for the formation of ordered aggregates in the form of amyloid fibrils [8,9]. The so-called “dialysis-related amyloidosis” (DRA) is, in fact, not related with the medical (dialysis) procedure itself; instead, it is a result of eliminating the life-threatening uremic states that exist before the hemodialysis treatment begins.

Amyloid fibril deposition in the organs and tissues accompanies several deleterious maladies, such as Alzheimer’s and Parkinson’s diseases, type II diabetes, prion diseases, and etc. However, recent studies let to the suggestion that in many cases the amyloid fibrils precursors, named amyloid oligomers, can be more toxic for cells than amyloid fibrils themselves [10,11,12,13]. Thereby, an assumption that amyloid fibrils are the result of these diseases rather than its cause [14] and their formation may even be a protective function of the organism appeared. However, in the case of DRA, the formation and accumulation of amyloid fibrils itself lead to a significantly reduction the patients life quality due to inflicted pain and the reduce of their (patients) mobility [15]. Most often, this disease appears in conjunction with the carpal tunnel syndrome, destructive spondyloarthropathy, atlantoaxial arthropathy, bursitis, bone cysts, pathological fractures, and other disorders [16,17,18,19,20,21,22]. In the later stages of DRA, amyloid plaques may eventually develop on the stomach and the heart walls. Solving the problem of DRA is currently given special attention because the number of patients in need of hemodialysis therapy increases every year. In the connection with the necessity of preventing β2M amyloid fibrils growth and accumulation, examination of these fibrils, especially their structure (that determine the fibrils cytotoxity and can be claimed in the development of ways to inhibit protein fibrillogenesis), became the aim of intensive research [23,24,25,26,27].

Recent studies of tissues and biological fluids of patients receiving long-term hemodialysis treatment revealed the existence of amyloid fibrils that are constructed not only from the full-length β2M (β2m), but also from its truncated forms that lack the 6 (ΔN6β2m) and 10 (ΔN10β2m) N-terminal amino acids of the polypeptide chain. The content of the truncated variants was about 25% [28,29,30]. A number of works showed that β2M amyloid fibrils that were prepared under different conditions [31,32] and formed from different variants of the protein [9,27,33] can form aggregates with different structure. The atomic-level structure of β2m and its truncated variant ΔN6β2m, early intermediates, and amyloid fibrils that were formed from these proteins were studied earlier by multidimensional magic angle spinning (MAS) NMR techniques and X-ray crystallography [26,34]. At the same time the structure of ΔN10β2m was not investigated till now.

The aim of the present work was the comparative study of the structural features of amyloid fibrils formed from the full-length and truncated forms of β2M while using the fluorescent probe thioflavin T (ThT). ThT became a gold standard in amyloid fibrils testing due to the specificity of its interaction with fibrils and a significant change of its fluorescence quantum yield while binding to the fibrils [35,36,37]. For investigation of ThT-β2M fibrils interaction a special technique was used in which the tested solutions were prepared via equilibrium microdialysis [38]. In addition, detected fluorescent characteristics of the samples were corrected on the primary inner filter effect (using a specially elaborated approach [39]), which was earlier not performed at all, or performed incorrectly, even by experienced specialists in spectroscopy. These methodical developments allowed for us to compare the binding parameters of ThT to amyloid fibrils formed from β2m and its truncated variants (ΔN6β2m and ΔN10β2m), characterize the photophysical properties of ThT bound to the examined amyloid fibrils and make new suggestions about these fibrils structure.

## 2. Results and Discussion

### 2.1. Different Morphology of Amyloid Fibrils Formed from Various Forms of β2M

**Figure 1:**
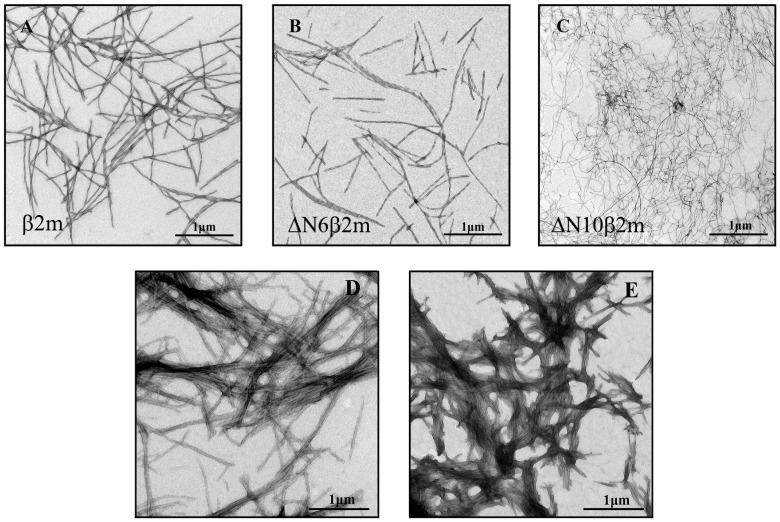
Electron micrographs of the amyloid fibrils formed from (**A**) beta-2-microglobulin (β2m), (**B**) ΔN6β2m, (**C**) ΔN10β2m, (**D**) insulin, and (**E**) lysozyme. Scale bar is 1 μm.

Amyloid fibrils were obtained on the basis of β2m, ΔN6β2m, and ΔN10β2m proteins created while using specific gene expression constructions (details of the experiment see in the Materials and Methods section). The morphology of prepared amyloid fibrils was evaluated by the use of electron microscopy (Figure 1A–C). The obtained images allowed for us to conclude that in vitro the truncated variants of β2M and the full-length protein form long, thin, straight amyloid fibrils with different morphology. In particular, the investigated samples differ in their diameter: β2m and ΔN6β2m amyloid fibril thickness is approximately 12–15 nm, and ΔN10β2m fibrils thickness is approximately 6–8 nm. The ΔN10β2m fibrils are more pliable and form loops and bends, which is possibly due to the small thickness, whereas fibrils formed from the protein with full-length amino acid sequence are more rigid and straight.

**Figure 2:**
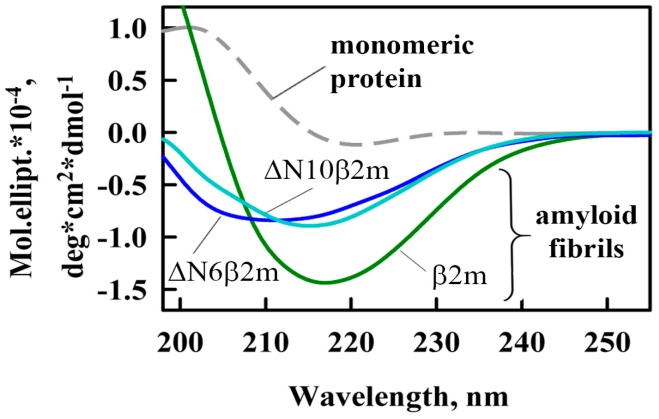
Far-UV CD spectra of β2m monomer (dashed gray curve) and amyloid fibrils formed from β2m (green curve), ΔN6β2m (blue curve) and ΔN10β2m (cyan curve).

Using the circular dichroism (CD) spectra registered in the far ultraviolet (UV) region the differences in the secondary structure of the investigated amyloid fibrils were shown (Figure 2). The most pronounced peak at about a wavelength of 220 nm (peak characteristic for proteins enriched with β-structure playing a key role in the formation of amyloid fibrils core) for β2m fibrils was observed. Thereby, the differences in the content of the β-structure observed by CD-spectroscopy indicate a different amount of amino acids in proteins that may be involved in their fibrillogenesis. This fact, in turn, can be a confirmation of the essential role of β2M N-terminal amino acids in the formation of amyloid fibrils.

**Figure 3:**
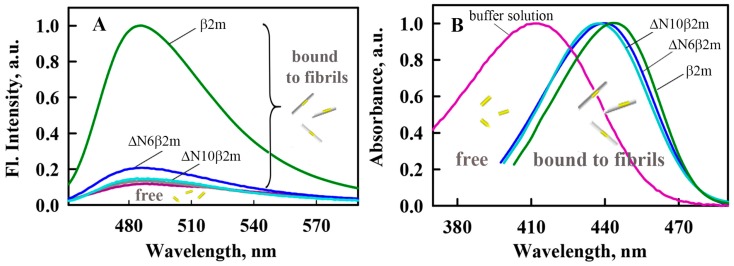
Spectral properties of thioflavin T (ThT) bound to β2M amyloid fibrils. (**A**) Fluorescence spectra of free ThT in water solution (purple curve) and the dye in the presence of β2m monomer (gray curve) and amyloid fibrils formed from β2m (green curve), ΔN6β2m (blue curve) and ΔN10β2m (cyan curve). (**B**) Absorption spectra of ThT bound to β2M amyloid fibrils determined with the use of solutions prepared by the equilibrium microdialysis. Absorption spectra of free ThT in water solution (purple curve) and the dye bound to β2m (green curve), ΔN6β2m (blue curve) and ΔN10β2m (cyan curve) amyloid fibrils are shown. All presented spectra normalized to unity at the spectral maxima.

To obtain information about the differences in the structure of β2M amyloid fibrils, their interaction with fluorescent probe thioflavin T (ThT) was also investigated. ThT specifically binds to amyloid fibrils and it is widely used as a test for their formation in a number of serious diseases, such as Alzheimer’s disease, Parkinson’s, and others. The essential feature of ThT is that in aqueous solution, the dye has a very low fluorescence quantum yield; however, when the dye binds to amyloid fibrils, this parameter may increase by several orders of magnitude [40,41]. In addition, the interaction of the dye with amyloid fibrils is very specific. Our results are consistent with these ideas for the β2m. We showed that the fluorescence intensity of ThT in the presence of monomeric β2m does not exceed the fluorescence intensity of the free dye in buffer solution and in the presence of β2m amyloid fibrils this value significantly increases (Figure 3A). At the same time, the fluorescence intensity of ThT bound to ΔN10β2m and ΔN6β2m fibrils is significantly lower than that of ThT bound to β2m fibrils (in the presence of ΔN10β2m amyloid fibrils fluorescence intensity of ThT practically does not change). These results on the one hand proved the differences of the amyloid fibrils formed from various forms of β2M and on the other hand showed the difficulties in their study with the use of ThT fluorescence. The last remark is important, since ThT fluorescent spectroscopy is widely used for determining the dye-fibrils binding parameters that can be used for analyzing of their structural features (see, for example, the review by Groenning [42]). In our previous works, we showed that for the solving of the problem of ThT-fibrils affinity and stoichiometry determination absorption spectroscopy of specially prepared by equilibrium microdialysis samples can be used [38,43,44].

### 2.2. Investigation of ThT-β2M Amyloid Fibrils Interaction Using Absorption Spectroscopy of Solutions Prepared by Equilibrium Microdialysis

First of all, it is necessary to note that the study of ThT-amyloid fibrils interaction and their binding parameters determination is complicated by the presence of an equilibrium system of free and fibril-associated ThT in the samples. Until recently, it was difficult to determine the characteristics of each of these dye fractions. As a solution for this problem, we proposed an approach based on use of equilibrium microdialysis for preparation of the investigated solutions [38]. This method was originally developed to study the interaction of low molecular weight ligands with their receptors; however, it has been undeservedly forgotten. In our recent works, we showed how this method (details of the experiment see in the Materials and Methods section) can be used for the determination of the ThT-amyloid fibrils binding parameters on the example of lysozyme and insulin fibrils [43,44]. It was assumed that the affinity and stoichiometry of the ThT binding to β2M amyloid fibrils could also be determined using this approach.

**Figure 4:**
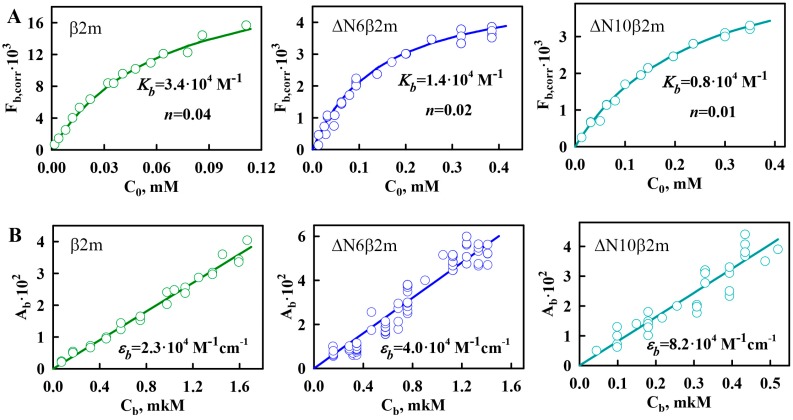

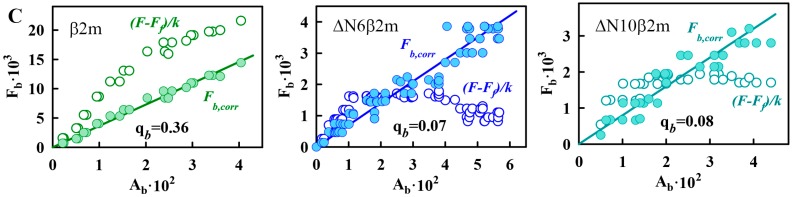
Investigation of β2M amyloid fibrils interaction with ThT. (**A**) Dependences of the fluorescence intensity of ThT bound to different variants of β2M amyloid fibrils corrected for the primary inner filter effect on the initial dye concentration. (**B**) Dependences of the absorbance of ThT bound to different variants of β2M amyloid fibrils on its concentration. (**C**) Dependences of the fluorescence intensity of the ThT bound to different variants of β2M amyloid fibrils, without (open circles) and with corrections (filled circles) for the primary inner filter effect on absorbance of bound dye. The panels (**A**–**C**) show the experimental data (circles), best-fit curves and lines (solid lines) and the determined values of the binding constants (*K*_b_) and the number of binding sites (*n*), the molar extinction coefficients ε_b_ and florescence quantum yields *q*_b_ of the ThT bound to β2m (left panels), ΔN6β2m (middle panels), and ΔN10β2m (right panels) amyloid fibrils.

Primarily, the solutions that were prepared by equilibrium microdialysis were used for the first time determination of the absorption spectrum of ThT incorporated into β2M amyloid fibrils. Obtained results show that the absorption spectra of ThT bound to β2m, ΔN6β2m and ΔN10β2m fibrils have a maximum at the wavelengths of 442, 441, and 438 nm, respectively (Figure 4B). The absorption spectrum of the free dye in aqueous solution is blue shifted (λ_max_ = 412 nm) [45], that indicates a significant interaction between the dye and polar solvent molecules [46]. At the same time, the absorption spectra of ThT incorporated into lysozyme and insulin amyloid fibrils (λ_max_ = 449–450 nm) are shifted to longer wavelengths in comparison to those of the absorption spectrum of the dye bound to β2M fibrils. We assume that upon binding to the β2M fibrils, the dye molecules are in a more polar microenvironment than when bound to other fibrils.

The results of the calculations indicate that after microdialysis for each type of β2M amyloid fibrils, the difference between the values of the initial concentration of ThT (introduced into the microdialysis chamber prior to equilibration) and the double concentration of free dye is comparable to the experimental error. This fact is apparently due to the low concentration of bound to β2m, ΔN6β2m, and ΔN10β2m fibrils dye. Therefore, the concentration of bound to fibrils dye (see the Equation (5) in the Materials and Methods section) and the ThT-β2M amyloid fibrils binding parameters cannot be determined while using absorption spectroscopy (see Equation (6) in Materials and Methods section). It was suggested that for this aim fluorescence spectroscopy of solutions prepared by equilibrium microdialysis could be used.

### 2.3. Determination of the ThT-β2M Amyloid Fibrils Binding Parameters Using Fluorescence Spectroscopy of Solutions Prepared by Equilibrium Microdialysis

The ThT fluorescence intensity in the presence of amyloid fibrils can be written, as follows:

(1) $$F=k^{\prime }I_{0}(1-10^{-A})\frac{\sum_{i}A_{\mathrm{FL},i}q_{\mathrm{FL},i}}{A}=kW\sum_{i}A_{\mathrm{FL},i}q_{\mathrm{FL},i}\;,$$

where *k*′ is a proportionality coefficient; *I*_0_ is the fluorescence intensity of the excitation light; $k=k^{\prime }I_{0}$ is a factor determined only by the experiment conditions; *A* is the total absorbance of the solution; $\left(1-10^{-A}\right)$ is a portion of the light absorbed by the solution; $W=\frac{1-10^{-A_{}}}{A_{}}$ is a correction factor that approaches a value of 2.303 at A→0; and, $A_{\mathrm{FL},i}$ and $q_{i}$ are the absorbance and fluorescence quantum yield of the fluorescent component *i*, respectively. The parameter *k* = *k*′*I*_0_ was determined from Equation (1) using the ATTO-425 fluorescent dye as an etalon sample [38]. ATTO-425 has spectral characteristics similar to that of ThT and a known value of fluorescence quantum yield.

Detection of the fluorescence spectra of the samples after equilibrium microdialysis showed that the fluorescence intensity of ThT in the presence of β2m fibrils (chamber #2) significantly exceeds the dye fluorescence in the absence of these amyloid fibrils (chamber #1). Thus, in the case of β2m fibrils, only the bound dye can be considered as a fluorescent component, and contribution of free dye fluorescence can be neglected. At the same time, the fluorescence intensity of ThT in the presence of ΔN6β2m and ΔN10β2m fibrils is comparable with the dye fluorescence in the absence of amyloid fibrils. Obtained results show that the use of equilibrium microdialysis is a key point in the samples preparation for the study of ThT interaction with ΔN6β2m and ΔN10β2m amyloid fibrils because of the large contribution of the free dye fluorescence intensity to the total fluorescence intensity (in contrast to situations in which the dye binding to fibrils is accompanied by a fluorescence intensity increase of several orders of magnitude, as in the case of β2m, lysozyme and insulin fibrils [43,44]).

To determine the true values of the fluorescence intensity of ThT bound to ΔN6β2m and ΔN10β2m amyloid fibrils corrected for the primary inner filter effect (*F*_b,corr_) [39] the contribution of the background fluorescence of the free dye (*F*_f_ = *kWA*_f_*q*_f_) to the recorded fluorescence intensity values of the ThT in the presence of the fibrils (*F* = *kW* (*A*_b_*q*_b_ + *A*_f_*q*_f_) = *kWA*_b_*q*_b_ + *F*_f_) was accounted, as follows:

(2) $$\frac{F-F_{f}}{kW}=F_{b,\mathrm{corr}}=A_{b}q_{b}.$$

To determine *F*_f_, the reference solutions that were prepared by equilibrium microdialysis were used (the free dye concentration in these solutions is equal to the concentration of free dye in the sample solutions). Using the Bouguer-Lambert-Beer law, the value of *A*_b_ can be represented as [38]:

(3) $$A_{b}=\varepsilon_{b}lC_{b}=\varepsilon_{b}l\frac{2+K_{b}n_{}C_{p}+K_{b}C_{0}-\sqrt{{\left(2+K_{b}nC_{p}+K_{b}C_{0}\right)}^{2}-4K_{b}^{2}nC_{p}C_{0}}}{2K_{b}},$$

where ε_b_ is the molar extinction coefficient of ThT bound to amyloid fibrils, *K*_b_ and *n* are ThT binding constant and number of binding sites to amyloid fibrils, *C*_p_ is a concentration of the protein that was used for the amyloid fibrils preparation, *C*_0_ is an initial concentration of ThT, and *l* is the optical path length (for details see Materials and Methods section and our previous work [38]). Thus, the dependence of the fluorescence intensity of the bound dye (corrected for the primary inner filter effect) on the initial ThT concentration was used to determine the binding parameters of ThT to amyloid fibrils:

(4) $$F_{b,\mathrm{corr}}=q_{b}\varepsilon_{b}l\frac{2+K_{b}n_{}C_{p}+K_{b}C_{0}-\sqrt{{\left(2+K_{b}nC_{p}+K_{b}C_{0}\right)}^{2}-4K_{b}^{2}nC_{p}C_{0}}}{2K_{b}}.$$

The experimental dependences of $F_{b,\mathrm{corr}}$ on *C*_0_ for ThT bound to β2m, ΔN6β2m, and ΔN10β2m amyloid fibrils are shown in Figure 4A. Parameters of ThT binding to β2M amyloid fibrils (binding constant and number of binding sites) were determined by multiple linear regressions. With the use of these binding parameters, the theoretical curves were plotted (Figure 4A). A poor approximation of the experimental data by the calculated curve may indicate that two or more binding modes exist and that Equation (4) cannot be used to determine the binding parameters. In the case of all investigated variants of β2M amyloid fibrils, linear approximation is satisfactory that demonstrates the reliability of the determined parameters and the correctness of the chosen model (in which all binding sites are equivalent and one binding mode of ThT-amyloid fibrils exists).

Despite the difference of the obtained binding constants of ThT to amyloid fibrils formed from β2m and its truncated forms, these parameters have the same order of magnitude (~10^−4^ M^−1^). It should be noted that ThT binding type with the similar values of affinity was previously found for other fibrils, for example, formed from insulin and lysozyme (Table 1). We believe that the detected binding mode of ThT to β2M and other amyloid fibrils corresponds to the incorporation of the dye into the grooves that are formed by the amino acids side chains of amyloid protofibrils, with the dye binding along the long axis of the fibrils that is perpendicular to the β-sheets (the model proposed by Krebs [47]). However, along with this binding mode for amyloid fibrils formed from insulin and lysozyme another binding type (with ThT binding constant ~10^−6^ M^−1^) was observed [43,44]. In order to make an assumption about the nature of the mode with high affinity in insulin and lysozyme fibrils and the reasons of its absence in β2M fibrils the morphology of these amyloid aggregates was compared. The electron microscopy data showed that, while fibrils on the basis of β2M (Figure 1A–C) are the long thin separate fibers, insulin and lysozyme fibrils could interact with each other forming clusters (Figure 2D,E). Therefore, we suggest that the existence of the second mode of ThT binding with higher binding constant may be caused by interaction of the dye with the areas of amyloid fibril clumping, which is not present in the case of β2M amyloid fibrils.

On the assumption that the first binding mode of ThT can be described by the Krebs model, the number of dye binding sites on the amyloid fibrils per protein molecule can be evaluated. А native β-sheet would need to contain at least five strands (strand-to-strand spacing ~4.7 Å, [48]) to be longer than a molecule of ThT (length ~15.2 Å). Monomers of some proteins can form two strands. This means that, to form a binding site for a ThT molecule, only 3–4 protein molecules may be enough. This is in agreement with the results obtained for the first mode of amyloid fibrils on the basis of lysozyme (Table 1) [43] and alpha-synuclein [49], which binding stoichiometry is about (1 ThT:4 protein) molecules. Some deviation from this proportion that was shown for insulin amyloid fibrils (~1:7) could be due to fibrils clumping and inaccessibility of binding sites in the depth of these clusters. It is interesting that the number of binding sites per protein molecule for the not clustered β2M fibrils is as follows: approximately 0.04 (i.e., one molecule of bound ThT accounted for 25 protein molecules), 0.02 (i.e., 1 ThT:50 protein molecules) and 0.01 (i.e., 1 ThT:100 protein molecules) for β2m, ΔN6β2m, and ΔN10β2m fibrils, respectively. It is possible to give several explanations for the observed ThT-β2M fibrils stoichiometry. It can be caused by the fact that potential binding sites are not available for the dye molecules due to the rigidity of β2M protofibrils interlacing (limiting the incorporation the dye into the binding site inside the fibrils wisp). It may be also associated with the “distortion” of the structure of potential sites for ThT incorporation as a result of the “twist” of protofibrils or the formation of bends of the fibrils wisp. These suggestions are in a good agreement with the correlation between the thicknesses of investigated fibrils (and also rigidity and straightness) shown using EM (Figure 1) and stoichiometry of ThT binding to β2M fibrils with different length of its N-terminal domain. It was concluded that ThT-fibrils binding parameters are diminished upon the shortening of the protein amino acid sequence and increasing the pliability (and decreasing the thickness) of the fibrils.

### 2.4. Photophysical Characteristics of ThT Bound to β2M Amyloid Fibrils

With the use of the determined values of ThT-fibrils binding parameters (*K*_b_ and *n*) and the absorbance of the bound dye (*A*_b_), the molar extinction coefficients of bound ThT were for the first time calculated (Table 1) while using Equation (5) (Figure 4B). The molar extinction coefficients of ThT bound to amyloid fibrils on the basis of different proteins and different types of β2M are significantly distinct and differ from the molar extinction coefficient of the free dye in aqueous solution (Table 1). It may be due to the differences in the dye conformation and microenvironment in various conditions.

Fluorescence quantum yield of the ThT bound to β2M fibrils (Table 1) was determined while using Equation (2). Figure 4C shows that the correction of the recorded values of the fluorescence intensity for the inner filter effect while using the coefficient *W* (which is only determined by the total absorbance of the solution) is a key detail in the use of the fluorescence approach and only after this correction fluorescence intensity numerically equals to the product of the absorbance and the fluorescence quantum yield of the object.

The determined fluorescence quantum yields of ThT bound to β2m, ΔN6β2m, and ΔN10β2m fibrils (~0.37, 0.07 and 0.08, respectively) are significantly higher than that of the free dye in aqueous solution (~0.0001) [40]. The fluorescence quantum yield of free ThT molecules is low because its benzothiazole and aminobenzene rings can rotate relative to one another in the excited state (which is typical for the molecular rotors that includes ThT), and the molecule can pass to the excited state with an angle between the planes of its rings that is close to 90°, which leads to the nonradiative transition of the dye to the ground state [46]. The significant increase in the fluorescence quantum yield of the dye when it binds to β2M amyloid fibrils is due to the restriction of the rotation of the dye fragments relative to one another in the excited state [40]. At the same time, the differences in fluorescence quantum yields of ThT that is bound to different types of β2M amyloid fibrils and other fibrils can be caused by varying degrees of dye fragments rotation restriction in the excited state (that is determined by the differences of ThT microenvironment).

**Figure 5:**
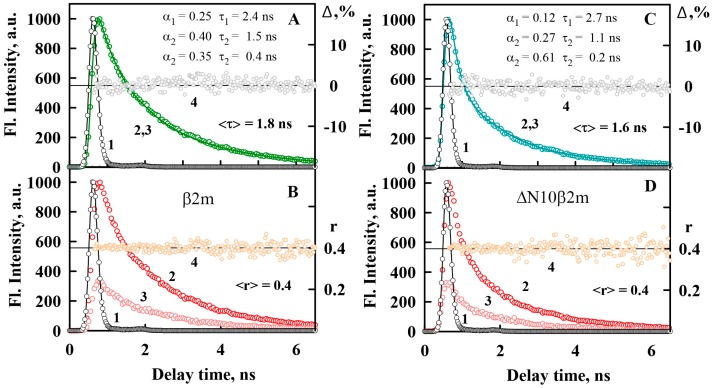
Time dependence of fluorescence of ThT bound to β2m (**A**,**B**) and ΔN10β2m (**C**,**D**) amyloid fibrils. (**A**,**C**) Fluorescence decay curves of the bound to fibrils dye. The excitation laser impulse profile (**1**), experimental fluorescence decay curve (**2**), best fit calculated fluorescence decay curve (**3**), and deviation between the experimental and calculated fluorescence decay curve (**4**) are shown. The fluorescence decay curve show best fit to a triexponential decay model. (**B**,**D**) Fluorescence anisotropy of the bound to fibrils ThT. The excitation laser impulse profile (**1**), the decay curves of the vertical (**2**) and horizontal (**3**) components of the fluorescence, and the time-dependent change in fluorescence anisotropy (**4**) over time are shown.

In the present work we also determined the values of ThT fluorescence lifetime in the presence of different types of β2M amyloid fibrils (Table 1). Figure 5A,C shows the dye fluorescence decay curves in the presence of β2m and ΔN10β2m amyloid fibrils, for which the greatest differences in the fluorescence lifetime were shown. Obtained values of the fluorescence lifetime of ThT bound to β2M amyloid fibrils are by three orders of magnitude greater than that for the dye in water solution (is about 1 ps [40,50]). Increase of the dye fluorescence lifetime accompanying its binding to amyloid fibrils can be caused by the restriction of the rotational motions of ThT fragments relative to each other in the excited state and by the decrease of the rate of the radiation-less deactivation of the dye molecules.

The observed differences in the fluorescence quantum yield and lifetime of the dye bound to β2M amyloid fibrils can be caused by the decrease of the microenvironment rigidity in the ThT binding sites upon the shortening of the protein amino acid sequence. These data are in accordance with the decrease in ThT-fibrils binding affinity at the same time and increase in fibrils pliability and thinning. All obtained results allow for demonstrating an essential role of N-terminal amino acids of the protein in the formation of the amyloid fibril core.

It was shown that the ThT fluorescence anisotropy in the presence of different types of β2M amyloid fibrils is the same and close to limiting value (Figure 5B,D). For the free dye in water solution a similar value of the fluorescence anisotropy was shown (~0.38) [50]. These results can be also explained by the molecular-rotor nature of ThT. In water solution relative rotation of the dye fragments relative to each other leads to radiation-less deactivation of the excited state of the dye molecules significantly faster than the molecule can change its spatial orientation (that in its turn can change ThT fluorescence anisotropy). When the dye binds to amyloid fibrils the characteristic time both of the mentioned above process increases and the fluorescence anisotropy of ThT remains the same high (Table 1). Thus, it was shown that the ThT fluorescence anisotropy (in contrast to other photophysical characteristics of this dye) is not sensitive to differences in the structure of amyloid fibrils and it can not be used for their detection and investigation, as it is suggested in some works [51].

## 3. Materials and Methods

### 3.1. Materials

The samples of “UltraPure Grade” ThT from AnaSpec (Fremont, CA, USA) were used without further purification. ThT was dissolved in 2 mM Tris-HCl buffer (pH 7.7) with 150 mM NaCl. All subsequent studies of the interaction of ThT with amyloid fibrils were performed strictly in the conditions in which the fibrils were obtained (in 150 mM Gly-HCl buffer (pH 2.5)). At the same time, a series of preliminary studies was made, which resulted in the conclusion that the properties of the dye in both of these buffers are identical. Fluorescent dye ATTO-425 from ATTO-TEC (Siegen, Germany) and the buffer components from Sigma (Louis, MO, USA) were used without additional purification. To determine the pH of tested solutions, HI 9024 pH meter (HANNA Instruments, Wensoket, RI, USA) was used.

### 3.2. Full-Length and Truncated Forms of β2M Expression and Purification 

To obtain soluble recombinant β2M and its truncated forms, special expression constructs were designed and *E.* Coli cells strain (DE3) were transformed with the appropriate vector. The protein synthesis was induced by adding isopropyl β-d-thiogalactoside (IPTG). The full-length β2m was obtained from the periplasmic space of the bacteria and it had no additional methionine at the N-terminus; instead, it began with isoleucine (first amino acid of human β2M), while the truncated forms ΔN6β2m and ΔN10β2m were obtained from the inclusion bodies and had methionine as their first N-terminus amino-acid. The proteins contained a polyhistidine sequence at the C-terminus, which made it possible to purify the protein very quickly and efficiently while using affinity chromatography on a nickel metal chelate-agarose sorbent. The yield of the fusion proteins was 30 mg of 1 L of bacterial culture. A more detailed description of the procedures of designing the vectors, expressing the proteins and their purification can be found in the Supplementary Materials. Obtained full-length and truncated forms of β2M were analyzed by electrophoretic separation in polyacrylamide gel electrophoresis under denaturing conditions and by the mass-spectral analysis.

### 3.3. Polyacrylamide Gel Electrophoresis

**Figure 6:**
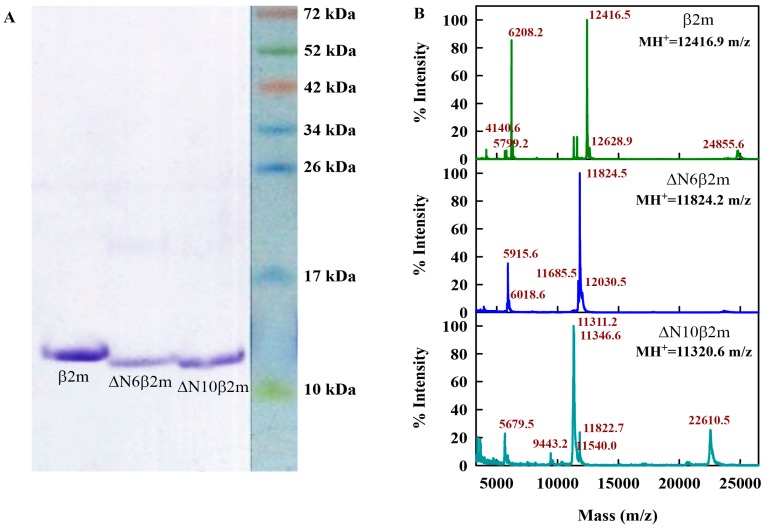
Characterization of the expressed and purified proteins. (**A**) Sodium dodecyl sulfate polyacrylamide gel electrophoresis (SDS-PAGE) of full-size β2m and their truncated variants: ΔN6β2m and ΔN10β2m. (**B**) Ion mass spectra of the β2m, ΔN6β2m, and ΔN10β2m. Peaks corresponding to main ion, double ion and dimer of main ion are seen for β2m and ΔN6β2m. For ΔN10β2m the main peak has complicated form, which can be the result of superposition of the one ion peak and double charged ion peaks of dimers or trimers of protein.

Electrophoretic analysis (EA) of proteins in gradient 5–15% of polyacrylamide gel electrophoresis (PAGE) in the presence 10% β-mercaptoethanol (Figure 6A) was performed according to standard procedure [52]. Staining of PAGE was performed while using Coomassie R-250 (Sigma-Aldrich, St. Louis, MO, USA).

### 3.4. Mass Spectral Analysis

Ion spectra were recorded using AB SCIEX TOF/TOF 5800 MALDI mass-spectrometer (AB Sciex, Framingham, MA, USA) in linear mode (Figure 6B). The instrument was calibrated using a Mass Standards Kit for Calibration of AB Sciex TOF/TOF Instruments (AB Sciex). Samples (0.5 µL) were spotted on a steel plate with 0.5 µL of sinapic acid matrix solution (Sigma-Aldrich) and air-dried at room temperature.

High-resolution mass spectra of the sample after trypsin digestion were recorded while using a Fourier Transform (Ion Cyclotron Resonance) Mass Spectrometer (Varian 902-MS, Palo Alto, CA, USA) equipped with MALDI and 9.4 T magnet (FTMS) in positive reflector mode. The instrument was calibrated while using ProteoMass Peptide MALDI-MS Calibration Kit (Sigma-Aldrich). The accuracy of the mass peak measurement was 2.5 ppm. Samples (0.5 µL) were spotted on a steel plate with 0.5 µL of a 2,5-Dihydroxybenzoic acid matrix (Sigma-Aldrich) and then air-dried at room temperature. In the spectra of trypsin digested recombinant protein ΔN6β2m, the peak corresponding to peptide MIQVYSR (mass 896.4658 Da) was recorded. That proves the presence of Met in the protein. However, because of the accuracy of the instrument, for trypsin digested recombinant protein ΔN10β2m, the peak corresponding to peptide (MSR (mass 393.192 Da)) has not been reliably detected.

### 3.5. Preparation of β2M Amyloid Fibrils

For the preparation of amyloid fibrils while using the full-length and truncated forms of β2M, isolated and purified proteins in concentration 30 μkM were incubated in Gly-HCl buffer (pH 2.5) in an incubator (37 °C) with constant agitation for 14 days (500 rpm) in a TS-100 Thermo-Shaker (Biosan, Warren, MI, USA).

### 3.6. Spectroscopic Studies

The absorption spectra were recorded while using a U-3900H spectrophotometer (Hitachi, Tokyo, Japan). The amyloid fibril absorption spectra were analyzed along with the light scattering using a standard procedure. The concentration of monomeric full-length protein and amyloid fibrils of β2m and ΔN6β2m was evaluated using a molar extinction coefficient of ε280 = 20,065 M^−1^ cm^−1^. The concentration of ΔN10β2m fibrils was evaluated while using a molar extinction coefficient of ε280 = 18,575 M^−1^ cm^−1^ because the sequence of ΔN10β2m is shorter by 10 amino acid residues and these residues include a tyrosine residue (Tyr10 in the full-length amino acid sequence). The path length of the cells used for absorption measurements was 0.5 cm.

CD spectra in the far UV-region were measured while using a J-810 spectropolarimeter (Jasco, Tokyo, Japan). The path length of CD cells was 0.1 cm. For all spectra, an average of three scans was obtained. The CD spectrum of the appropriate buffer was recorded and subtracted from the sample spectra.

Fluorescence spectra and fluorescence excitation spectra were measured using a Cary Eclipse spectrofluorimeter (Varian, Sydney, Australia). Fluorescence intensity was corrected to the primary inner filter effect, as described earlier [39]. Fluorescent dye ATTO-425, whose fluorescence and absorption spectra are similar to that of ThT, was taken as a reference for determining the corrected and normalized values of fluorescence intensity of ThT bound to amyloid fibrils. The fluorescence quantum yield of ATTO-425 is 0.9. The path length of the cells that were used for fluorescence spectra and fluorescence excitation spectra measurements was 0.5 cm.

For all spectroscopic studies, the amyloid fibrils in concentration about 0.4 mg/mL were used.

### 3.7. Electron Microscopy

To obtain electron micrographs, the method of negative staining with a 1% aqueous solution of uranyl acetate was used. Amyloid fibrils in concentration 1 mg/mL were placed on copper grids that were coated with a collodion film-substrate. Transmission electron microscope Libra 120 (Carl Zeiss, Jena, Germany) was used to obtain the images.

### 3.8. Equilibrium Microdialysis

The Harvard Apparatus/Amika (Holliston, MA, USA) device for equilibrium microdialysis consisting of two chambers (500 μL each) that were separated by a membrane was used for the sample preparation (for more details see [38]). The separating membrane was impermeable to particles larger than 10,000 Da. Amyloid fibrils (the receptor) were placed in the buffer solution at concentration *C*_p_ (concentration of the protein that is used to prepare the amyloid fibrils) in chamber #1 and ThT (the ligand) was placed in the same buffer and at an initial concentration *C*_0_ in chamber #2. After the equilibration the concentrations of free dye in chambers #1 and #2 became equal (*C*_f_), whereas the total ThT concentration in chamber #1 was more than that in chamber #2 by the concentration of bound dye (*C*_b_). Thus, the method of equilibrium microdialysis allowed us to prepare the sample and reference solutions that were used for the determination of the absorption spectrum of ThT bound to amyloid fibrils and the concentrations *C*_f_ and *C*_b_. Given that chambers #1 and #2 have identical volumes Equation (5) can be written:

*C*_b_ = *C*_0_ − 2 *C*_f_. (5)

The binding constants (*K*_b*i*_) and the number of ThT binding sites (*n_i_*) for the various binding modes of amyloid fibrils (*i*) can be calculated using the Equation:

(6) $$C_{b}=\sum_{i}\frac{n_{i}C_{p}C_{f}}{K_{di}+C_{f}}=\sum_{i}\frac{n_{i}C_{p}C_{f}K_{bi}}{1+C_{f}K_{bi}},$$

where $K_{di}=\frac{1}{K_{bi}}$ is a dissociation constant.

### 3.9. Time-Resolved Fluorescence Measurements 

FluoTime 300 spectrometer (Pico Quant, Berlin, Germany) with the Laser Diode Head LDH-C-440 (λex = 440 nm) was used to record the fluorescence decay curves in the subnanosecond and nanosecond range. With the use of the standard convolute-and-compare nonlinear least-squares procedure [53] the measured emission decays were fitted to a multiexponential function. Comparison of the convolution of the model exponential function with the instrument response function to the experimental data until a satisfactory fit is obtained was carried out. Cross correlation of the excitation and the fundamental gate pulse was applied to measure the instrument response function (IRF). The nonlinear least-squares method underlies the fitting routine. Minimization was performed according to Marquardt [54]. We add the definition for “IRF”, please confirm.

Fluorescence anisotropy was determined as: $r=\left(I_{V}^{V}-GI_{H}^{V}\right)/\left(I_{V}^{V}+2GI_{H}^{V}\right)$, where $I_{V}^{V}$ and $I_{H}^{V}$ are vertical and horizontal components of fluorescence intensity excited by vertical polarized light, and $G=I_{V}^{H}/I_{H}^{H}$ is the coefficient that determines the different sensitivity of the registering system for vertical and horizontal components of fluorescence intensity.

## 4. Conclusions

In the present work, the interaction of β2M amyloid fibrils with the specific fluorescent probe ThT was investigated with the use of the solutions that were prepared by equilibrium microdialysis. By the absorption spectroscopy of the obtained samples the differences in the absorption spectra of the dye bound to fibrils formed from full-length protein (β2m) and its truncated forms that lack the 6 (ΔN6β2m) and 10 (ΔN10β2m) N-terminal amino acids were shown for the first time. The results of this work prove that fluorescence spectroscopy is a more sensitive method than absorption spectroscopy for determination of ThT-β2M amyloid fibrils binding parameters. It was noted that, in the case of amyloid fibrils that were formed from the truncated forms of β2M, preparation of the tested solutions while using the equilibrium microdialysis method is a key point for this aim. It is caused by the necessity of accounting of the portion of free dye fluorescence (which is considerable in the case of ΔN6β2m and ΔN10β2m fibrils) in the total recorded fluorescence intensity. In our work, we show how this problem can be solved correctly with the use of the reference solutions prepared by the proposed approach. In addition in the present work correction of the recorded values of ThT fluorescence intensity on the primary inner filter effect was performed that in the most works is either not performed at all, or is performed incorrectly. Thus, the use of special methodological developments allowed us to determine the affinity and stoichiometry of ThT interaction with β2M amyloid fibrils as correctly as it is possible. It should be noted that the binding parameters for ThT with ΔN10β2m fibrils were determined for the first time.

The existence of one binding mode (binding type) of the dye molecules to different variants of β2M amyloid fibrils was shown. This mode can be caused by the incorporation of the dye into the grooves formed by the amino acids side chains of amyloid protofibrils, with the dye binding along the long axis of the fibrils that is perpendicular to the β-sheets. Using the corrected on the primary inner filter effect fluorescence intensity and absorption of solutions that were prepared by equilibrium microdialysis, fluorescence quantum yields of ThT bound to β2M fibrils were calculated. Their contrast to values for the dye in aqueous solution was explained by the molecular rotor nature of ThT molecules.

At the same time the obtained results show that a relatively small increase in the fluorescence intensity of the dye bound to protein aggregates does not always indicate the absence of amyloid fibrils in the sample. This fact, according to Equation (4), can be determined by the relatively low affinity and stoichiometry of dye binding to fibrils, and also by the relatively low molar extinction coefficient and fluorescence quantum yield of the bound dye. Listed factors are characteristic for one of the ThT binding types, which can usually be found in amyloid fibrils formed from various amyloidogenic proteins, and, in particular, in β2M amyloid fibrils. However, for example, in insulin and lysozyme fibrils, another type of ThT binding can be found as well, which determines a significant increase in fluorescence intensity of the bound dye [38,43].

The observed differences in the determined ThT-β2M fibrils binding parameters and characteristics of the bound dye allowed for proving, not only the difference of the ΔN10β2m fibrils from other β2M fibrils (that can be detected visually, for example, by transmission electron microscopy (TEM)), but also the differences between β2m and ΔN6β2m fibrils (that were not obvious from TEM). The observed decrease in the affinity and stoichiometry of ThT interaction with β2M fibrils, as well as in the fluorescence quantum yield and lifetime of the bound dye upon shortening of the protein amino acid sequence, suggests an essential role of N-terminal amino acids of the protein in the formation of the amyloid fibril. Information about amyloidogenic protein sequences can be claimed in the development of ways to inhibit β2M fibrillogenesis for the treatment of dialysis-related amyloidosis.

The obtained results prove that the methodological developments presented by us can be used as a sensitive tool for investigation of amyloid fibrils polymorphism. This is especially important when it is necessary to take into account the contribution of photophysical characteristics of free ThT to the total photophysical characteristics of the sample, as, for example, in the case of fibrils formed from truncated forms of β2M. The proposed approach can be further used, for example, for a comparative study of the structure of amyloid fibrils on the basis of the full-length and truncated forms of β2M, with the structure of “geterofibrils” [34], as well as the structure of the fibrils formed from these proteins under the influence of various external factors (for examle, aB-crystallin [25]).

## Figures and Tables

**Table 1:** Binding parameters of ThT to amyloid fibrils formed from different amyloidogenic proteins and characteristics of the bound dye.

Object	Binding Mode	λ_max_, nm	*K*_b*i*_ × 10^−5^, M^−1^	*n_i_*	ε*_i_* *10^−4^, M^−1^cm^−1^	*q_i_*	*<τ>*, ns	*r*
ThT + β2m amyloid fibrils	1	442 ± 1	0.34 ± 0.04	0.041 ± 0.006	2.3 ± 0.3	0.36 ± 0.03	1.80 ± 0.03	0.40 ± 0.01
ThT + ΔN6β2m amyloid fibrils	1	441 ± 1	0.14 ± 0.03	0.020 ± 0.004	4.0 ± 0.4	0.07 ± 0.02	1.66 ± 0.03	0.39 ± 0.01
ThT + ΔN10β2m amyloid fibrils	1	438 ± 1	0.08 ± 0.03	0.009 ± 0.004	8.2 ± 0.5	0.08 ± 0.03	1.62 ± 0.03	0.40 ± 0.01
ThT + insulin amyloid fibrils [44]	1	449	0.35	0.14	2.3	0.27	-	-
	2	448	78	0.02	7.9	0.72	-	-
ThT + lysozyme amyloid fibrils [43]	1	451	0.60	0.25	6.2	0.0001	-	-
	2	449	72	0.11	5.3	0.44	-	-
ThT free in water solution [40]	-	412	-	-	3.2	0.0001	0.001	0.38

## Supplementary Materials

Supplementary materials can be found at http://www.mdpi.com/1422-0067/19/9/2762/s1. Table S1: The amino acid sequence of the full-length and truncated forms of β2M; Figure S1: Principle of equilibrium microdialysis experiment.

## Abbreviations

β2M	beta-2-microglobulin
β2m	full-length β2M
N6β2m	β2M truncated form that lacks the 6 N-terminal amino acids of the polypeptide chain
N10β2m	β2M truncated form that lacks the 10 N-terminal amino acids of the polypeptide chain
DRA	dialysis-related amyloidosis
ThT	thioflavin T
TEM	transmission electron microscopy
CD	circular dichroism
UV	ultraviolet
IPTG	isopropyl β-d-thiogalactoside
SDS-PAGE	sodium dodecyl sulfate polyacrylamide gel
IRF	instrument response function
