# Correction: Sulatskaya et al. Structural Features of Amyloid Fibrils Formed from the Full-Length and Truncated Forms of Beta-2-Microglobulin Probed by Fluorescent Dye Thioflavin T. *Int. J. Mol. Sci.* 2018, *19*, 2762

**DOI:** 10.3390/ijms25126411

**Published:** 2024-06-11

**Authors:** Anna I. Sulatskaya, Natalia P. Rodina, Dmitry S. Polyakov, Maksim I. Sulatsky, Tatyana O. Artamonova, Mikhail A. Khodorkovskii, Mikhail M. Shavlovsky, Irina M. Kuznetsova, Konstantin K. Turoverov

**Affiliations:** 1 Laboratory of Structural Dynamics, Stability and Folding of Proteins, Institute of Cytology of the Russian Academy of Science, Tikhoretsky ave. 4, St. Petersburg 194064, Russia; ansul@mail.ru (A.I.S.);; 2 Department of Molecular Genetics, Institute of Experimental Medicine, Pavlov str. 12, St. Petersburg 197376, Russia; 3 Chair of Medical Genetics, North-Western State Medical University named after I.I. Mechnikov, Piskarevskij prospect 47, St. Petersburg 195067, Russia; 4 Research Center of Nanobiotechnologies, Peter the Great St. Petersburg Polytechnic University, Polytechnicheskaya 29, St. Petersburg 195251, Russia; 5 Institute of Physics, Nanotechnology and Telecommunications, Peter the Great St. Petersburg Polytechnic University, Polytechnicheskaya 29, St. Petersburg 195251, Russia

In the original publication [1], some representative images of different amyloids published by us earlier [2] were used for a comparison with the main objects of study [1]. The citation to our previous publication has now been inserted into the caption of Figure 1 in [1] and should read: “Electron micrographs of the amyloid fibrils formed from (**A**) beta-2-microglobulin (β2m), (**B**) ΔN6β2m, (**C**) ΔN10β2m, (**D**) insulin, and (**E**) lysozyme. (**A**,**D**,**E**) are adapted from [40]. Scale bar is 1 μm.”. With this correction, the order of other references has been adjusted accordingly.

The authors state that the scientific conclusions are unaffected. This correction was approved by the Academic Editor. The original publication has also been updated.
