# Supplementary material for: Structural Features of Amyloid Fibrils Formed from the Full-Length and Truncated Forms of Beta-2-Microglobulin Probed by Fluorescent Dye Thioflavin T
Source: Int J Mol Sci. 2018 Sep 14;19(9):2762. doi: 10.3390/ijms19092762 (PMC6164334; doi:10.3390/ijms19092762)
Supplement: Supplementary file 1 [file ijms-19-02762-s001.pdf]

Supplementary materials

Structural Features of Amyloid Fibrils Formed From the Full-length and Truncated Forms of Beta-2-microglobulin Probed by Fluorescent Dye Thioflavin T

Anna I. Sulatskaya [1], Natalia P. Rodina [1], Dmitry S. Polyakov [2,3], Maksim I. Sulatsky [1], Tatyana O. Artamonova [4], Mikhail A. Khodorkovskii [4], Mikhail M. Shavlovsky [2,3], Irina M. Kuznetsova [1] and Konstantin K. Turoverov [1,5,*]

[1] Laboratory of Structural Dynamics, Stability and Folding of Proteins, Institute of Cytology of the Russian Academy of Science, Tikhoretsky ave. 4, St. Petersburg 194064, Russia; ansul@mail.ru (A.I.S.); natalia240994@gmail.com (N.P.R.); m_sulatsky@mail.ru (M.I.S.); imk@incras.ru (I.M.K.)
[2] Department of Molecular Genetics, Institute of Experimental Medicine, Pavlov str. 12, St. Petersburg 197376, Russia; ravendoctor@mail.ru (D.S.P.); mmsch@rambler.ru (M.M.S.)
[3] Chair of Medical Genetics, North-Western State Medical University named after I.I. Mechnikov, Piskarevskij prospect 47, St. Petersburg 195067, Russia
[4] Research and Innovation Complex "Nanobiotechnologies", Peter the Great St.-Petersburg Polytechnic University, Polytechnicheskaya 29, St. Petersburg 195251, Russia; artamonova@nanobio.spbstu.ru (T.O.A.); khodorkovskii@mail.ru (M.A.K.)
[5] Institute of Physics, Nanotechnology and Telecommunications, Peter the Great St. Petersburg Polytechnic University, Polytechnicheskaya 29, St. Petersburg 195251, Russia
* Correspondence: kkt@incras.ru; Tel.: +7-812-297-19-57

1. Full-length and Truncated Forms of β2M Expression and Purification

To obtain soluble recombinant beta-2-microglobulin (β2M), the vector pETb2m6.8 was created. The vector was designed in such way that β2M is synthesized with the bacterial PelB "leader peptide" transporting the protein to the periplasmic space of the bacteria and subsequently cleaved by the action of bacterial proteases. This method provided β2m without an additional methionine at the N-terminus; instead, it began with isoleucine (first amino acid of human β2m) (Table S 1).

Table S1. The amino acid sequence of the full-length and truncated forms of β2M.

**Native Human β2m**	10 20 30 40 50<br>IQRTPKIQVY SRHPAENGKS NFLNCYVSGF HPSDIEVDLL KNGERIEKVE<br>60 70 80 90 99<br>HSDLSFSKDW SFYLLYYTEF TPTEKDEYAC RVNHVTLSQP KIVKWDRDM
**Recombinant β2m**	10 20 30 40 50<br>IQRTPKIQVY SRHPAENGKS NFLNCYVSGF HPSDIEVDLL KNGERIEKVE<br>60 70 80 90 100<br>HSDLSFSKDW SFYLLYYTEF TPTEKDEYAC RVNHVTLSQP KIVKWDRDMH<br>104<br>HHHH
**Recombinant ΔN6β2m**	10 20 30 40 50<br>-----MIQVY SRHPAENGKS NFLNCYVSGF HPSDIEVDLL KNGERIEKVE<br>60 70 80 90 100<br>HSDLSFSKDW SFYLLYYTEF TPTEKDEYAC RVNHVTLSQP KIVKWDRDMH<br>104<br>HHHH
**Recombinant ΔN10β2m**	10 20 30 40 50<br>---------M SRHPAENGKS NFLNCYVSGF HPSDIEVDLL KNGERIEKVE<br>60 70 80 90 100<br>HSDLSFSKDW SFYLLYYTEF TPTEKDEYAC RVNHVTLSQP KIVKWDRDMH<br>104<br>HHHH

Recombinant full-length β2M (β2m) was prepared from *E*.coli cells strain BL21 (DE3) transformed with the pETb2m6.8 vector. *E*.coli cells were grown overnight in 10 ml of Luria-Bertani (LB) medium, containing 1 mg of ampicillin, and after were introduced into 500 ml of fresh medium. The cultivation was continued at 37 °C under aeration conditions until the optical density of A (600) = 0.6–1.0. β2m synthesis was induced by adding IPTG to a final concentration of 0.5 mM, and subsequent cultivation overnight at 26 °C under aeration conditions. After deposition of the bacterial cells by centrifugation for 25 min at 10,000 g, the periplasmic fraction was obtained by the "osmotic shock" method [1].

As expected, soluble β2m was found in the periplasm. The protein contained a polyhistidine sequence at the C-terminus, which made it possible to purify the protein very quickly and efficiently using affinity chromatography on a nickel metal chelate-agarose sorbent (the volume of column was 1.5 mL). For this aim, phenylmethylsulfonyl fluoride and imidazole (final concentration 1 mM and 10 mM, respectively) were added to the resulting contents of the bacterial periplasm. The solution was filtered through a metal chelate sorbent. The ballast proteins were successively washed with 0.5 m NaCl solution in 0.1 M K-phosphate buffer, pH = 8.0, containing 20–30 to 40 mM imidazole. Further, the protein was eluted with 0.15 M NaCl, pH = 7.4, containing 200 mM imidazole. The extraction of the periplasmic fractions and the subsequent affinity chromatography yielded β2m with a purity greater than 98%.

To obtain β2M without the 6 (ΔN6β2m) and 10 (ΔN10β2m) N-terminal amino acids of the polypeptide chain (Table S1), gene expression constructs were created. Instead of the PelB "leader peptide", the start codon ATG was inserted before the nucleotide sequence encoding ΔN6β2m and ΔN10β2m in a bacterial plasmid. As a result, the protein synthesized from the resulting gene expression construct accumulated in inclusion bodies and had an additional methionine (due to the start codon) at the N-terminus.

The vector for obtaining recombinant human β2m without the 6 N-terminal amino acids (ΔN6β2m) was constructed using forward primer 5'catatgattcaggtttactcacgt3' [2] and backward primer 5'gtcaagcttatcagtgatggtgatggtgcatgtctcgatcccactt3'. As a template during the PCR a plasmid comprising full-length wild type β2m was used [3]. The vector for the production of recombinant human β2m without the 10 N-terminal amino acids (ΔN10β2m) was constructed similarly, but the forward primer had the sequence: 5'ctgcatatgtcacgtcatccagcaga3'. The PCR products were treated with restriction enzymes NdeI and HindIII (Fermentas) and inserted into the plasmid pET22b (+) (Novagen), treated with appropriate restriction enzymes.

Recombinant ΔN6β2m and ΔN10β2m were prepared from E.coli cells strain BL21 (DE3), transformed with the appropriate vector. *E*.coli cells were grown overnight in 10 mL of Luria-Bertani (LB) medium, containing 1 mg of ampicillin, and after were introduced into 500 mL of fresh medium. The cultivation was continued at 37° C under aeration conditions until the optical density of A (600) = 0.6-1.0. Protein synthesis was induced by adding IPTG to a final concentration of 0.5 mM, and subsequent cultivation overnight at 37° C under aeration conditions. After deposition of the bacterial cells by centrifugation for 25 minutes at 10 000 g, the cells were once washed with saline (PBS) followed by precipitation at 10 000 g. To the laundered cell pellet PBS was added and cells were disrupted by sonication (3 kHz frequency range 3 to 30 s.) in cooling conditions adding water with ice. For a more complete destruction 1/5 volume of glass beads was added to a suspension (Glass Beads, 500 microns, Sigma).

For obtaining initial soluble proteins, β2M truncated forms, renaturation directly on the affinity column was carried out. For this purpose, the inclusion bodies, after washing up from bacterial lysate, were dissolved in 8 M urea in the presence of mercaptoethanol. After separation of insoluble aggregates by centrifugation, the supernatant was supplied to a nickel agarose column. The urea was washed with buffer with decreasing concentration of urea. Complete removal of urea was achieved by washing the column with 0.15 M sodium chloride with 30 mM imidazole. Proteins were eluted by 200 mM imidazole solution. The elution of proteins was tested by absorbance of the eluate at 280 nm. Imidazole was removed from preparations of soluble protein (monomer) by ultrafiltration through membranes retaining a material with a molecular weight more than 5 kD. The yield of the fusion proteins was 30 mg of 1 liter of bacterial culture.

Obtained the full-length and truncated forms of β2M were analyzed by electrophoretic separation in polyacrylamide gel electrophoresis under denaturing conditions and by the mass-spectral analysis.

2. Principle of Equilibrium Microdialysis Experiment

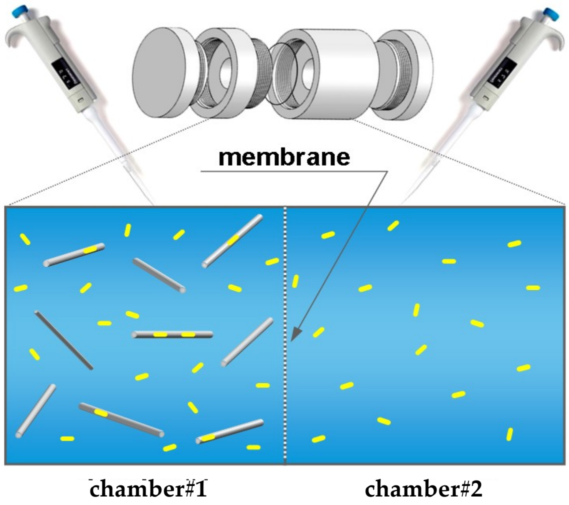

Figure S1. Principle of equilibrium microdialysis experiment [4,5]. Equilibrium microdialysis was performed using a Harvard Apparatus/Amika (USA) device that consists of two chambers (500 μL each) that are separated by a membrane that is impermeable to particles larger than 10,000 Da. Equilibrium microdialysis implies the allocation of two interacting agents, a ligand and receptor, in two chambers (#2 and #1, respectively) that are divided by a membrane permeable to the ligand and impermeable to the receptor. The essence of the method is as follows: amyloid fibrils are placed in the buffer solution at concentration C_P (concentration of the protein that is used to prepare the amyloid fibrils) in chamber #1, and the dye is placed in the same buffer and at an initial concentration C_0 in chamber #2. After equilibration, the free ThT concentrations in chambers #1 and #2 become equal (C_f), whereas the total ThT concentration in chamber #1 exceeds that in chamber #2 by the concentration of bound dye (C_b). Thus, using the method of equilibrium microdialysis, sample and reference solutions can be prepared; these solutions can be used to determine the absorption spectrum of the ThT bound to amyloid fibrils and the concentrations C_f and C_b.

References

1. Ausubel, F.M.; Brent, R.; Kingston, R.E.; Moore, D.D.; Seidman, J.G.; Smith, J.A.; Struhl, K. Current Protocols in Molecular Biology. John Wiley & Sons: New York, NY, USA, 1989.
2. Esposito, G.; Michelutti, R.; Verdone, G.; Viglino, P.; Hernandez, H.; Robinson, C.V.; Amoresano, A.; Dal Piaz, F.; Monti, M.; Pucci, P.; et al. Removal of the N-terminal hexapeptide from human beta2-microglobulin facilitates protein aggregation and fibril formation. *Protein science : a publication of the Protein Society* **2000,** *9*, 831–845.
3. Solovyov, K.V.; Polyakov, D.S.; Grudinina, N.A.; Egorov, V.V.; Morozova, I.V.; Aleynikova, T.D.; Shavlovsky, M.M. Expression in E. coli and purification of the fibrillogenic fusion proteins TTR-sfGFP and beta2M-sfGFP. *Prep Biochem Biotechnol* **2011,** *41*, 337–349.
4. Kuznetsova, I.M.; Sulatskaya, A.I.; Uversky, V.N.; Turoverov, K.K. A new trend in the experimental methodology for the analysis of the thioflavin T binding to amyloid fibrils. *Molecular neurobiology* **2012,** *45*, 488–498.
5. Sulatskaya, A.I.; Kuznetsova, I.M.; Turoverov, K.K. Interaction of thioflavin T with amyloid fibrils: stoichiometry and affinity of dye binding, absorption spectra of bound dye. *The journal of physical chemistry. B* **2011,** *115*, 11519–11524.
